# Conceptual views of mental health among adolescents in Sweden

**DOI:** 10.1093/eurpub/ckac129.055

**Published:** 2022-10-25

**Authors:** N Durbeej, AC Karlsson, A Sarkadi

**Affiliations:** Department of Public Health and Caring Sciences, Uppsala University, Uppsala, Sweden

## Abstract

**Background:**

Lay and professional people may use terms for mental health and mental health problems differently, causing difficulties in adequately addressing associated needs. Despite the public health issue of increased mental health problems among adolescents, there is limited research on perceptions of mental health concepts among young people. This study aimed to explore conceptual views of mental health and mental health problems among adolescents.

**Methods:**

During October and November 2020, a total of 32 adolescents (15-18 years old) living on Sweden's largest island Gotland were interviewed in focus groups or individual interviews. The interviews were semi-structured and audio recorded. Data were analysed thematically according to Systematic Text Condensation.

**Results:**

Three themes emerged from the analysis: Mental health is about how we feel; One's mental health depends on one's situation, thoughts and ways of coping; and Mental health problems should be taken seriously and can get severe. The adolescents described mental health as an overarching concept encompassing both positive mental health and mental health problems. Mental health problems were perceived as something other than normal challenges in life, however ranging from minor problems to severe illness. Good mental health was understood as a condition with absence of mental health problems and presence of symptoms of positive mental health.

**Conclusions:**

The adolescents’ had a complex and holistic understanding of mental health concepts, consistent with definitions used by the World Health Organization and Swedish authorities. They suggested both positive mental health and mental health problems to be considered when assessing and discussing their mental health. Further, the results highlight the need of support for young people on how to cope with difficulties in life and support for those suffering from minor mental health problems.

**Key messages:**

• The adolescents’ understanding of mental health and mental health problems were highly consistent with current accepted definitions of the concepts.

• According to the participants, both positive mental health and mental health problems should be considered simultaneously to understand and address adolescents’ mental health.

